# Short-Chain Fatty Acids (SCFAs) Modulate the Hepatic Glucose and Lipid Metabolism of *Coilia nasus* via the FFAR/AMPK Signaling Pathway In Vitro

**DOI:** 10.3390/ijms26083654

**Published:** 2025-04-12

**Authors:** Jun Gao, Qi Mang, Yi Sun, Gangchun Xu

**Affiliations:** 1 Freshwater Fisheries Research Center, Chinese Academy of Fishery Sciences, Wuxi 214081, China; gaojun@ffrc.cn (J.G.); sunyi@ffrc.cn (Y.S.); 2 Wuxi Fisheries College, Nanjing Agriculture University, Wuxi 214081, China; mangq@cafs.ac.cn

**Keywords:** *Coilia nasus*, SCFAs, FFAR/AMPK, high glucose, high lipid

## Abstract

The expansion of intensive aquaculture has heightened metabolic dysregulation in fish caused by high-glucose and high-lipid (HG-HL) diets, contributing to growth retardation and hepatic pathologies. Using *Coilia nasus* hepatocytes, this study investigated the regulatory effects of short-chain fatty acids (SCFAs) on glucose-lipid metabolism. In vitro HG-HL exposure elevated intracellular glucose, triglycerides (TG), and cholesterol; suppressed catalase (CAT) and superoxide dismutase (SOD); and dysregulated metabolic genes (upregulated *phosphoenolpyruvate carboxykinase* and *acetyl-CoA carboxylase*; downregulated *glucokinase* and *hormone-sensitive lipase*). Co-treatment with acetate and propionate reversed these anomalies, reducing TG and cholesterol, restoring antioxidant capacity (SOD and CAT), and normalizing gene expression patterns. Molecular docking suggested potential binding interactions between SCFAs and free fatty acid receptor (FFAR2/3). This study provided initial evidence suggesting SCFAs might attenuate HG-HL-induced metabolic stress in a teleost model, potentially involving FFAR-related pathways and AMPK-associated responses. The findings contribute to understanding SCFA-mediated metabolic regulation in fish, offering preliminary support for developing dietary interventions to manage aquacultural metabolic syndromes.

## 1. Introduction

Short-chain fatty acids (SCFAs) are products of the anaerobic fermentation of indigestible carbohydrates by the gut microbiota. In aquatic animals, the predominant SCFAs are acetate, propionate, and butyrate, with acetate and propionate constituting a significant proportion. These compounds play a crucial role in modulating host metabolism [1]. Although, carbohydrates and lipids can reduce the consumption of protein as an energy source (a phenomenon known as protein sparing) [2]. However, excessive levels of carbohydrates and lipids in the feed can trigger adverse reactions such as oxidative stress, mitochondrial dysfunction, and metabolic disorders in the organism, leading to a decline in the growth performance of fish and even causing death [3]. Currently, SCFAs are widely applied in aquaculture as green and safe additives. The addition of appropriate amounts of sodium acetate, sodium propionate, and sodium butyrate in feed can promote the growth of species such as the beluga sturgeon (*Huso huso*), Caspian whitefish (*Rutilus frisii kutum*), and grass carp (*Ctenopharyngodon idellus*) [4,5,6]. Moreover, among the physiological functions of SCFAs, the regulation of glucose and lipid metabolism has attracted the attention of the majority of researchers.

The liver is a crucial tissue for glucose metabolism. The regulation of blood glucose homeostasis encompasses two aspects: the production and consumption of blood glucose. SCFAs phosphorylate AMP-activated protein kinase (AMPK) and its downstream target gene acetyl-CoA carboxylase (ACC). Butyrate increases the phosphorylation of AMPK in mice fed a high-fat diet [7]. SCFAs directly activate AMPK by elevating the AMP/ATP ratio [8]. The activation of AMPK inhibits glycogen and protein synthesis while promoting glucose transport and fatty acid oxidation [9]. In the liver, AMPK activation reduces the gene expression of key gluconeogenic enzymes such as glucose-6-phosphatase (*g6pase*) and phosphoenolpyruvate carboxykinase (*pepck*) [10]. SCFAs directly activate the AMPK signaling pathway by increasing the AMP/ATP ratio in both muscle and liver [11,12]. Furthermore, AMPK can influence numerous downstream signaling pathways, such as the mammalian target of rapamycin (mTOR) and peroxisome proliferator-activated receptor gamma coactivator 1-alpha (PGC-1α) [13,14].

Lipid metabolism is a key process regulated by SCFAs. As members of the fatty acid family, SCFAs provide substrates for lipid synthesis. Sodium acetate and sodium butyrate are the primary synthetic lipid matrices in the colonic epithelial cells of rats, capable of converting SCFAs into acetyl-CoA [15]. Similarly, the gut microbiota promotes hepatic fatty acid metabolism by providing high levels of acetate as a precursor for the synthesis of palmitate and stearate [16]. Acetyl-CoA can not only enter the tricarboxylic acid (TCA) cycle to generate energy but also produce palmitic acid under the action of cytosolic enzyme systems. These cytosolic enzyme systems can be translocated to the mitochondria to elongate carbon chains and form triglycerides with other substances stored in adipose tissue. SCFAs not only participate in lipid metabolism as substrates but also act as regulatory factors that modulate lipid metabolism. Butyrate increases the oxidation of fatty acids in brown adipose tissue and improves diet-induced obesity and insulin resistance [17]. In addition to glucose metabolism, the AMPK signaling pathway also plays a role in lipid metabolism. Activation of the AMPK signaling pathway in mice increases the expression of peroxisome proliferator-activated receptor α (PPARα) and p-ACC in the liver and reduces the levels of triglycerides and free fatty acids [18]. Furthermore, hormone-sensitive lipase (HSL) and adipose triglyceride lipase (ATGL), as the primary enzymes of lipolysis, are regulated by AMPK. Studies have shown that the activated AMPK signaling pathway can promote the expression of *hsl* and *atgl* and enhance lipolysis [19,20]. SCFAs also regulate lipid metabolism by inhibiting intracellular lipolysis, thereby reducing the plasma levels of free fatty acids. Furthermore, the activation of AMPK signaling in rat liver cancer cells suppresses the synthesis of hepatic fatty acids by inhibiting sterol regulatory element-binding protein 1C (SREBP-1C) [21].

Previous studies have indicated that intervention with composite probiotics can improve the metabolism of the gut microbiota in *C. nasus*, with increased levels of acetate and propionate that are significantly correlated with hepatic glucose and lipid metabolism genes [22]. Therefore, this study investigated the regulatory relationship between these SCFAs and hepatic glucose and lipid metabolism genes by treating primary cultured hepatocytes of *C. nasus* with acetic acid and propionic acid. Additionally, molecular docking analysis was employed to explore the interaction between acetic acid and propionic acid and free fatty acid receptors.

## 2. Results

### 2.1. Effects of High Glucose on the Hepatocytes of C. nasus

*C. nasus*-derived hepatocytes maintained >90% viability (Trypan Blue exclusion) and displayed characteristic spherosomes under phase-contrast microscopy, confirming successful in vitro expansion (see Supplementary Materials Figure S1). As shown in Figure S2A (see Supplementary Materials), the cell viability of hepatocytes under 20 and 25 mmol/L glucose significantly decreased compared to other groups (*p* < 0.05). Therefore, 25 mmol/L glucose was used as the high glucose group in subsequent experiments.

**Figure 1:**
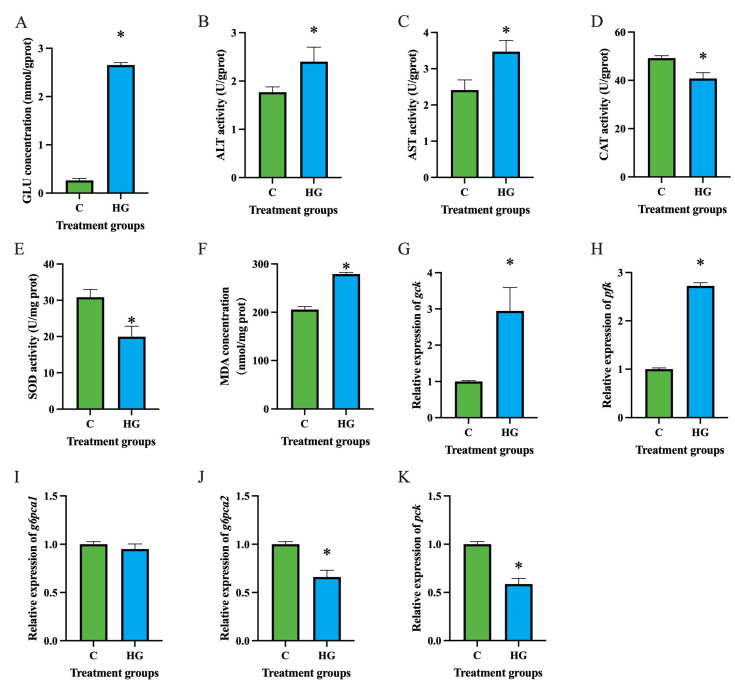
Effects of high glucose on glucose metabolism in hepatocytes of *Coilia nasus*. Glucose concentration (**A**), alanine aminotransferase (**B**), aspartate aminotransferase (**C**), catalase (**D**), superoxide dismutase (**E**), malondialdehyde (**F**), *gck* (**G**), *pfk* (**H**), *g6pca1* (**I**), *g6pca2* (**J**), and *pck* (**K**). Results were presented as means ± SE. Asterisks indicate significant differences (*p* < 0.05). C: control group; HG: high glucose group. GLU: glucose, ALT: alanine aminotransferase, AST: aspartate aminotransferase, CAT: catalase, SOD: superoxide dismutase, MDA: malondialdehyde, *gck*: glucokinase, *pfk*: phosphofructokinase, *g6pca*: glucose-6-phosphatase catalytic subunit, *pck*: phosphoenolpyruvate carboxykinase.

After co-incubation with 25 mmol/L glucose for 24 h, the glucose content in hepatocytes of the high-glucose group significantly increased (Figure 1A). The activities of alanine aminotransferase (ALT) (Figure 1B) and aspartate aminotransferase (AST) (Figure 1C) in hepatocytes significantly increased after high glucose treatment (*p* < 0.05), while the activities of catalase (CAT) (Figure 1D) and superoxide dismutase (SOD) (Figure 1E) significantly decreased (*p* < 0.05). Additionally, the malondialdehyde (MDA) level in hepatocytes significantly increased after high glucose treatment (*p* < 0.05) (Figure 1F). The mRNA levels of glycolysis-related genes glucokinase (*gck*) (Figure 1G) and phosphofructokinase (*pfk*) (Figure 1H) in hepatocytes significantly increased (*p* < 0.05), whereas the mRNA levels of gluconeogenesis-related genes *g6pca2* (Figure 1J) and phosphoenolpyruvate carboxykinase (*pck*) (Figure 1K) significantly decreased after high glucose treatment (*p* < 0.05).

### 2.2. Effects of High Lipid on the Hepatocytes of C. nasus

As shown in Figure S2B (see Supplementary Materials), the cell viability of hepatocytes under 1.0 mmol/L oleic acid significantly decreased compared to other groups (*p* < 0.05). Therefore, 1.0 mmol/L oleic acid was used as the high lipid group in subsequent experiments.

**Figure 2:**
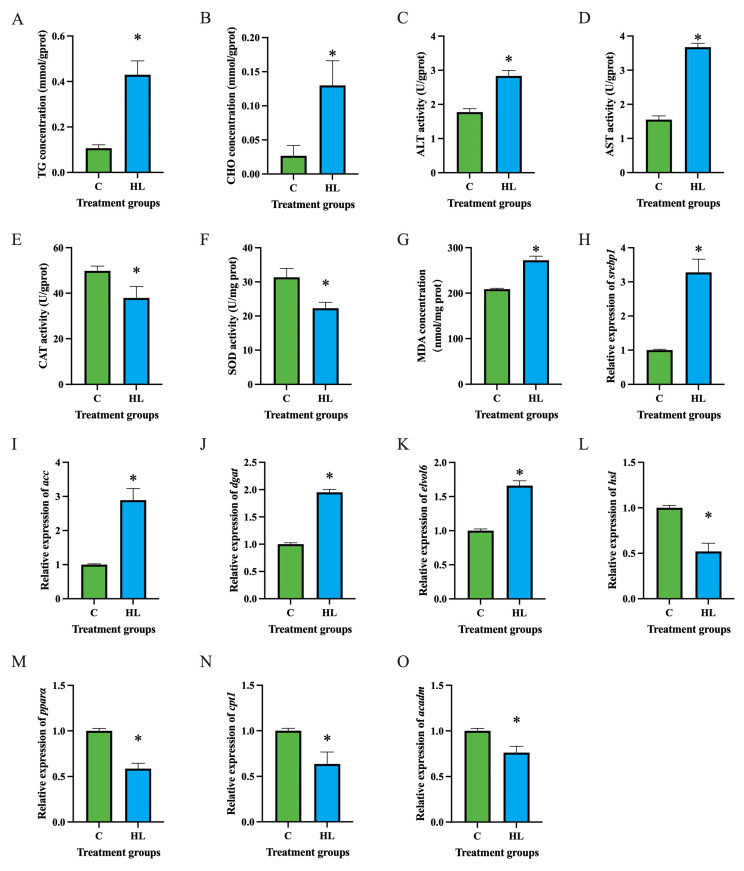
Effects of high lipid on lipid metabolism in hepatocytes of *C. nasus*. Triglycerides (**A**), cholesterol (**B**), alanine aminotransferase (**C**), aspartate aminotransferase (**D**), catalase (**E**), superoxide dismutase (**F**), malondialdehyde (**G**), *srebp1* (**H**), *acc* (**I**), *dgat* (**J**), *elvol6* (**K**), *hsl* (**L**), *pparα* (**M**), *cpt1* (**N**), and *acadm* (**O**). Results were presented as means ± SE. Asterisks indicate significant differences (*p* < 0.05). C: control group; HL: high lipid group. *srebp1*: sterol-regulatory element binding protein 1, *acc*: acetyl-coa carboxylase, *dgat*: diacylglycerol acyltransferase, *elvol6*: elongation of very long-chain fatty acids protein 6, *hsl*: hormone-sensitive lipase, *pparα*: peroxisome proliferators-activated receptors, *cpt1*: carnitine palmitoyltransferase 1, *acadm*: acyl-coa dehydrogenase.

After co-incubation with 1.0 mmol/L oleic acid for 24 h, the contents of triglyceride (TG) (Figure 2A) and cholesterol (CHO) (Figure 2B) in hepatocytes of the high lipid group significantly increased (*p* < 0.05). The activities of ALT (Figure 2C) and AST (Figure 2D) in hepatocytes significantly increased after high lipid treatment (*p* < 0.05), while the activities of CAT (Figure 2E) and SOD (Figure 2F) significantly decreased (*p* < 0.05). Additionally, the MDA level in hepatocytes significantly increased after high lipid treatment (*p* < 0.05) (Figure 2G). The mRNA levels of lipid synthesis-related genes *srebp1* (Figure 2H), *acc* (Figure 2I), diacylglycerol acyltransferase (*dgat*) (Figure 2J), and elongation of very long-chain fatty acids protein 6 (*elvol6*) (Figure 2K) in hepatocytes significantly increased (*p* < 0.05); whereas the mRNA levels of lipid catabolism-related genes *hsl* (Figure 2L), *pparα* (Figure 2M), carnitine palmitoyltransferase 1 (*cpt1*) (Figure 2N), and acyl-coa dehydrogenase (*acadm*) (Figure 2O) significantly decreased after high lipid treatment (*p* < 0.05).

### 2.3. Effects of Short-Chain Fatty Acids on Hepatocytes of C. nasus Treated with High Glucose

After co-incubating hepatocytes with high glucose (25 mmol/L glucose) and 0, 2, 4, 6, and 8 mmol/L acetic acid and propionic acid for 24 h, the viability of hepatocytes from *C. nasus* increased after treatment with 2, 4, 6, and 8 mmol/L acetic acid, but there were no significant differences among the groups (*p* > 0.05) (Supplementary Materials Figure S3A). Additionally, as shown in Figure S3B (see Supplementary Materials), the viability of hepatocytes from *C. nasus* increased after treatment with 2, 4, 6, and 8 mmol/L propionic acid, but there were no significant differences among the groups (*p* > 0.05).

**Figure 3:**
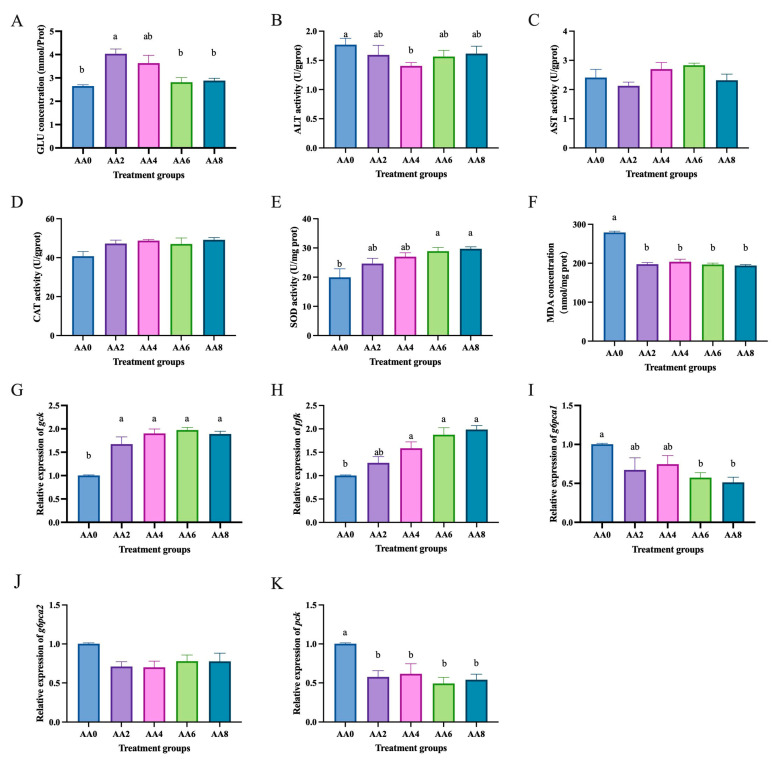
Effects of acetic acid on glucose metabolism in hepatocytes of *C. nasus* induced by high glucose. Glucose concentration (**A**), alanine aminotransferase (**B**), aspartate aminotransferase (**C**), catalase (**D**), superoxide dismutase (**E**), malondialdehyde (**F**), *gck* (**G**), *pfk* (**H**), *g6pca1* (**I**), *g6pca2* (**J**), and *pck* (**K**). Results were presented as means ± SE. Different letters indicate a significant difference (*p* < 0.05). AA: acetic acid.

Compared to the high glucose group (0 mmol/L acetic acid), the group treated with acetic acid (2 mmol/L) showed a significant increase in glucose concentration (*p* < 0.05) (Figure 3A). The group treated with acetic acid (4 mmol/L) had significantly lower ALT activity than the high glucose group (*p* < 0.05) (Figure 3B). The groups treated with acetic acid (6 and 8 mmol/L) had significantly higher SOD activity than the high glucose group (*p* < 0.05) (Figure 3E). The groups treated with acetic acid (2, 4, 6, and 8 mmol/L) had significantly lower MDA levels than the high glucose group (*p* < 0.05) (Figure 3F). Moreover, there were no significant differences in AST (Figure 3C) and CAT (Figure 3D) activities among the groups (*p* > 0.05). Compared to the high glucose treatment group, the acetic acid treatment groups significantly increased the mRNA levels of glycolysis-related genes *gck* (Figure 3G) and *pfk* (Figure 3H) (*p* < 0.05) and significantly decreased the mRNA levels of gluconeogenesis-related genes *g6pca1* (Figure 3I) and *pck* (Figure 3H) (*p* < 0.05).

**Figure 4:**
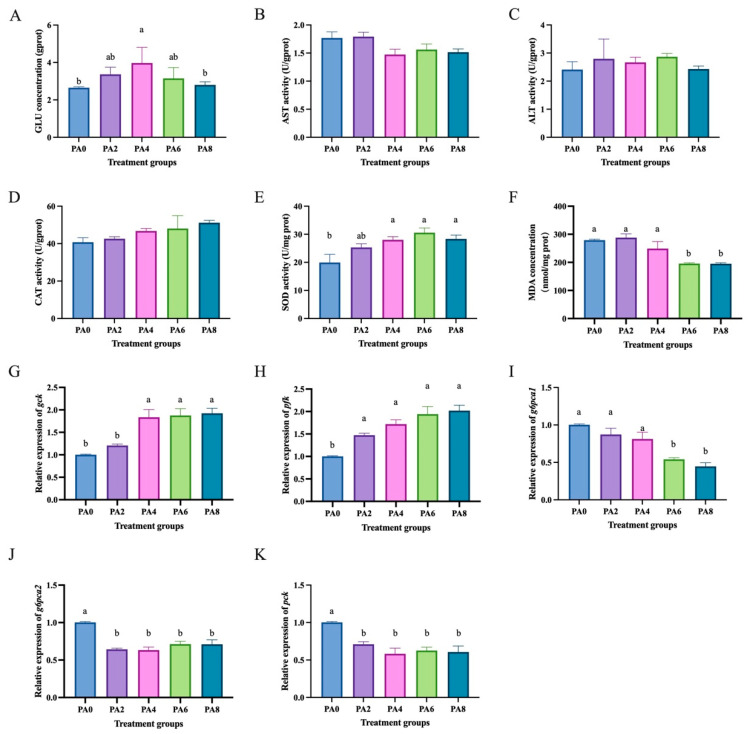
Effects of propionic acid on glucose metabolism in hepatocytes of *C. nasus* induced by high glucose. Glucose concentration (**A**), alanine aminotransferase (**B**), aspartate aminotransferase (**C**), catalase (**D**), superoxide dismutase (**E**), malondialdehyde (**F**), *gck* (**G**), *pfk* (**H**), *g6pca1* (**I**), *g6pca2* (**J**), and *pck* (**K**). Results were presented as means ± SE. Different letters indicate a significant difference (*p* < 0.05). PA: propionic acid.

Compared to the high glucose group (0 mmol/L propionic acid), the group treated with propionic acid (4 mmol/L) showed a significant increase in glucose concentration (*p* < 0.05) (Figure 4A). The groups treated with propionic acid (4, 6, and 8 mmol/L) had significantly higher SOD activity than the high glucose group (*p* < 0.05) (Figure 4E). The groups treated with propionic acid (6 and 8 mmol/L) had significantly lower MDA levels than the high glucose group (*p* < 0.05) (Figure 4F). Moreover, there were no significant differences in ALT (Figure 4B), AST (Figure 4C), and CAT (Figure 4D) activities among the groups (*p* > 0.05). Additionally, compared to the high glucose treatment group, the propionic acid treatment groups significantly increased the mRNA levels of glycolysis-related genes *gck* (Figure 4G) and *pfk* (Figure 4H) (*p* < 0.05) and significantly decreased the mRNA levels of gluconeogenesis-related genes *g6pca1* (Figure 4I), *g6pca2* (Figure 4J), and *pck* (Figure 4K) (*p* < 0.05).

### 2.4. Effects of Short-Chain Fatty Acids on Hepatocytes of C. nasus Treated with High Lipids

After co-incubating hepatocytes with high lipid (1.0 mmol/L oleic acid) and 0, 2, 4, 6, and 8 mmol/L acetic acid and propionic acid for 24 h, the viability of hepatocytes from *C. nasus* increased after treatment with 2, 4, 6, and 8 mmol/L sodium acetate and sodium propionate, but there were no significant differences among the groups (*p* > 0.05) (Supplementary Materials Figure S4).

**Figure 5:**
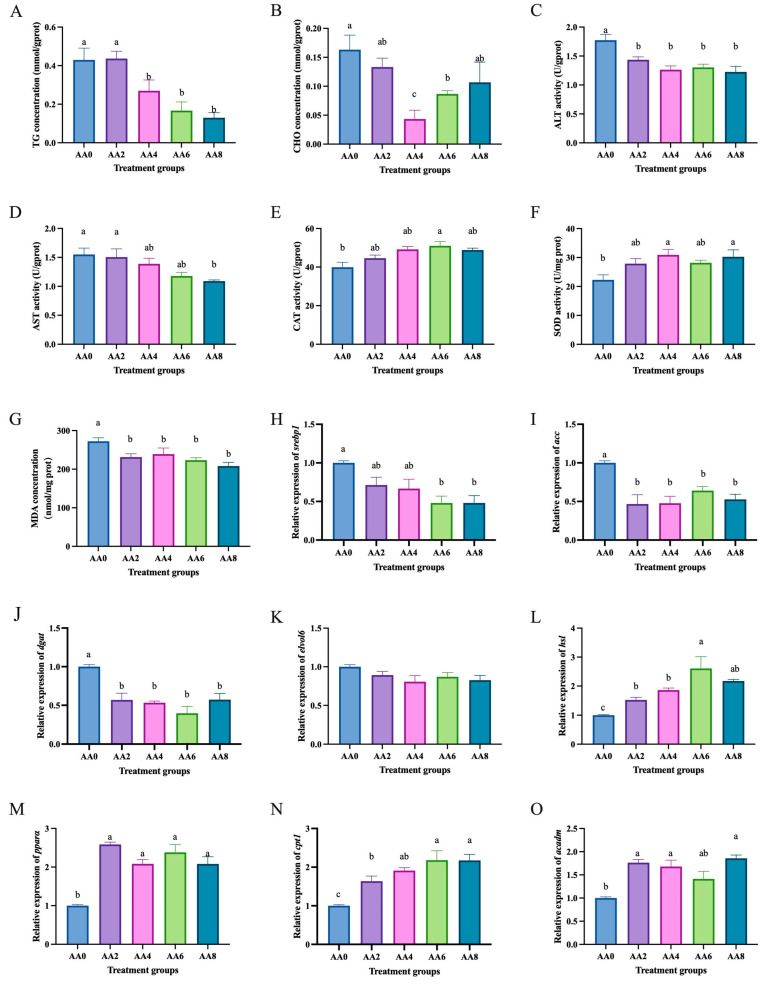
Effects of acetic acid on lipid metabolism in hepatocytes of *C. nasus* induced by high lipids. Triglycerides (**A**), cholesterol (**B**), alanine aminotransferase (**C**), aspartate aminotransferase (**D**), catalase (**E**), superoxide dismutase (**F**), malondialdehyde (**G**), *srebp1* (**H**), *acc* (**I**), *dgat* (**J**), *elvol6* (**K**), *hsl* (**L**), *pparα* (**M**), *cpt1* (**N**), and *acadm* (**O**). Results were presented as means ± SE. Different letters indicate a significant difference (*p* < 0.05). AA: acetic acid.

Compared to the high lipid group (0 mmol/L acetic acid), the group treated with acetic acid (4, 6, and 8 mmol/L) had significantly lower triglyceride levels (*p* < 0.05) (Figure 5A), and the group treated with acetic acid (4 and 6 mmol/L) had significantly lower cholesterol levels (*p* < 0.05) (Figure 5B). The group treated with acetic acid had significantly lower ALT activity than the high-lipid group (*p* < 0.05) (Figure 5C), and the group treated with acetic acid (8 mmol/L) had significantly lower AST activity than the high-lipid group (*p* < 0.05) (Figure 5D). The group treated with acetic acid (6 mmol/L) had significantly higher CAT activity than the high-lipid group (*p* < 0.05) (Figure 5E), and the groups treated with acetic acid (4 and 8 mmol/L) had significantly higher SOD activity than the high-lipid group (*p* < 0.05) (Figure 5F). The group treated with acetic acid had significantly lower MDA levels than the high-lipid group (*p* < 0.05) (Figure 5G). Compared to the high-lipid treatment group, the acetic acid treatment groups significantly decreased the mRNA levels of lipid synthesis-related genes *srebp1* (Figure 5H), *acc* (Figure 5I), and *dgat* (Figure 5J) (*p* < 0.05) and significantly increased the mRNA levels of lipid catabolism-related genes *hsl* (Figure 5L), *pparα* (Figure 5M), *cpt1* (Figure 5N), and *acadm* (Figure 5O) (*p* < 0.05).

**Figure 6:**
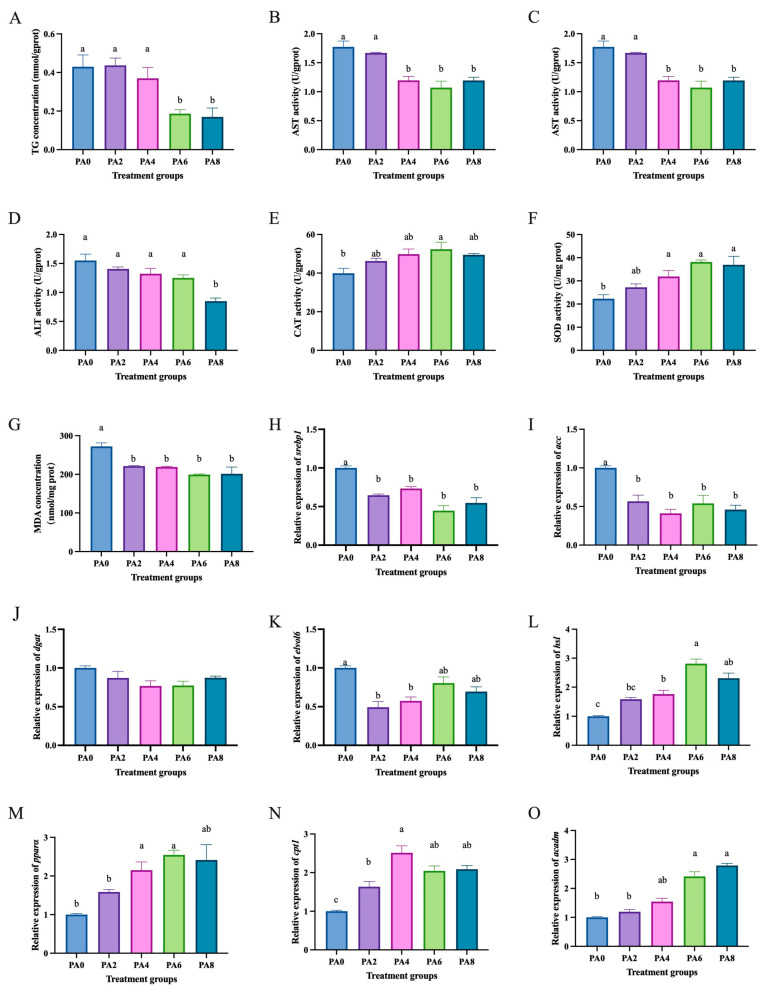
Effects of propionic acid on lipid metabolism in hepatocytes of *C. nasus* induced by high lipids. Triglycerides (**A**), cholesterol (**B**), alanine aminotransferase (**C**), aspartate aminotransferase (**D**), catalase (**E**), superoxide dismutase (**F**), malondialdehyde (**G**), *srebp1* (**H**), *acc* (**I**), *dgat* (**J**), *elvol6* (**K**), *hsl* (**L**), *pparα* (**M**), *cpt1* (**N**), and *acadm* (**O**). Results were presented as means ± SE. Different letters indicate a significant difference (*p* < 0.05). PA: propionic acid.

Compared to the high lipid group (0 mmol/L propionic acid), the group treated with propionic acid (6 and 8 mmol/L) had significantly lower TG levels (*p* < 0.05) (Figure 6A), and the group treated with propionic acid (6 mmol/L) had significantly lower CHO levels (*p* < 0.05) (Figure 6B). The groups treated with propionic acid (4, 6, and 8 mmol/L) had significantly lower ALT activity than the high-lipid group (*p* < 0.05) (Figure 6C), and the group treated with propionic acid (8 mmol/L) had significantly lower AST activity than the high-lipid group (*p* < 0.05) (Figure 6D). The group treated with propionic acid (6 mmol/L) had significantly higher CAT activity than the high-lipid group (*p* < 0.05) (Figure 6E), and the groups treated with propionic acid (4, 6, and 8 mmol/L) had significantly higher SOD activity than the high-lipid group (*p* < 0.05) (Figure 6F). The group treated with propionic acid had significantly lower MDA levels than the high-lipid group (*p* < 0.05) (Figure 6G). Moreover, compared to the high-lipid treatment group, the propionic acid treatment groups significantly decreased the mRNA levels of lipid synthesis-related genes *srebp1* (Figure 6H), acc (Figure 6I), and elvol6 (Figure 6K) (*p* < 0.05) and significantly increased the mRNA levels of lipid catabolism-related genes *hsl* (Figure 6L), *pparα* (Figure 6M), *cpt1* (Figure 6N), and *acadm* (Figure 6O) (*p* < 0.05).

### 2.5. Molecular Docking Results of SCFAs with Free Fatty Acid Receptor (FFAR)

This study employed molecular docking methods to investigate the interaction between FFAR and SCFA. Homology modeling was first conducted for FFAR2 and FFAR3. The Ramachandran plot score for the FFAR2 model was 95.13% (see Supplementary Materials Figure S4A), and the QMEAN local scores were 0.60 (see Supplementary Materials Figure S4B). For the FFAR3 model, the Ramachandran plot score was 96.30% (see Supplementary Materials Figure S4C), and the QMEAN local scores were 0.61 (see Supplementary Materials Figure S4D). Both models had Ramachandran plot scores above 90% and QMEAN local scores greater than 0.6, indicating good quality of the modeled structures.

**Figure 7:**
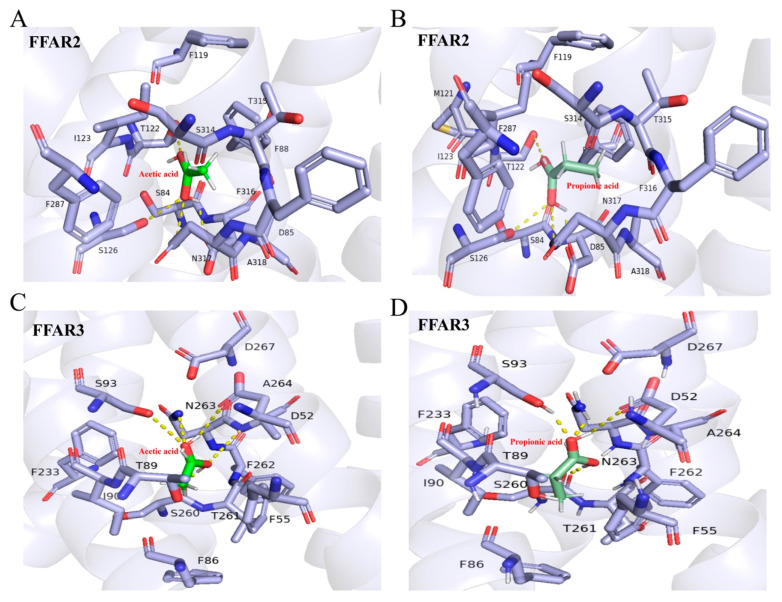
Interface interaction analysis of free fatty acid receptors (FFARs) and short chain fatty acids (SCFAs). (**A**) 3D diagrams of the interface interaction between FFAR2 and acetic acid complex; (**B**) 3D diagrams of the interface interaction between FFAR2 and propionic acid complex; (**C**) 3D diagrams of the interface interaction between FFAR3 and acetic acid complex; (**D**) 3D diagrams of the interface interaction between FFAR3 and complex.

The docking results between FFAR2 and acetic acid (AA) are shown in Table S2 (see Supplementary Materials), with the highest binding score of −8.18 kcal/mol, primarily maintained through van der Waals forces and hydrogen bonds (see Supplementary Materials Table S4). The specific binding mode is depicted in Figure 7A. The docking results between FFAR2 and propionic acid (PA) are shown in Table S3 (see Supplementary Materials), with the highest binding score of −9.39 kcal/mol, primarily maintained through van der Waals forces and hydrogen bonds (see Supplementary Materials Table S5). The specific binding mode is depicted in Figure 7B. The docking results between FFAR3 and acetic acid (AA) are shown in Table S6 (see Supplementary Materials), with the highest binding score of −8.06 kcal/mol, primarily maintained through van der Waals forces and hydrogen bonds (see Supplementary Materials Table S8). The specific binding mode is depicted in Figure 7C. The docking results between FFAR3 and propionic acid (PA) are shown in Table S7, with the highest binding score of −9.52 kcal/mol, primarily maintained through van der Waals forces and hydrogen bonds (see Supplementary Materials Table S9). The specific binding mode is depicted in Figure 7D.

### 2.6. Expression of ffar2, ffar3, and ampkα Treated with SCFAs and High Glucose and Lipid in Hepatocytes of C. nasus

**Figure 8:**
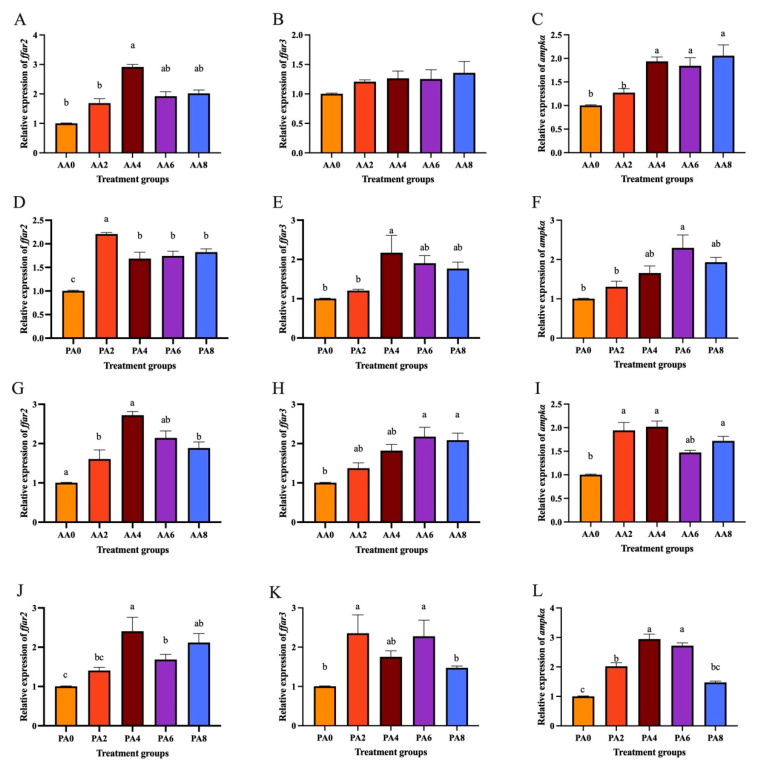
Effects of acetic acid on expression of *ffar2* (**A**), *ffar3* (**B**), and *ampkα* (**C**) in hepatocytes of *C. nasus* induced by high glucose. Effects of propionic acid on expression of *ffar2* (**D**), *ffar3* (**E**), and *ampkα* (**F**) in hepatocytes of *C. nasus* induced by high glucose. Effects of acetic acid on expression of *ffar2* (**G**), *ffar3* (**H**), and *ampkα* (**I**) in hepatocytes of *C. nasus* induced by high lipids. Effects of propionic acid on expression of *ffar*2 (**J**), *ffar3* (**K**), and *ampkα* (**L**) in hepatocytes of *C. nasus* induced by high lipids. Results were presented as means ± SE. Different letters indicate a significant difference (*p* < 0.05). AA: acetic acid; PA: propionic acid.

Compared to the high glucose treatment group, the acetic acid treatment groups significantly increased the mRNA levels of *ffar2* (Figure 8A) and *ampkα* (Figure 8C) (*p* < 0.05). Moreover, the propionic acid treatment groups also increased the mRNA levels of *ffar2* (Figure 8D), *ffar3* (Figure 8E), and *ampkα* (Figure 8F) (*p* < 0.05).

Compared to the high-lipid treatment group, the acetic acid treatment groups significantly increased the mRNA levels of *ffar2* (Figure 8G), *ffar3* (Figure 8H), and *ampkα* (Figure 8I) (*p* < 0.05). Moreover, the propionic acid treatment groups also increased the mRNA levels of *ffar2* (Figure 8J), *ffar3* (Figure 8K), and *ampkα* (Figure 8L) (*p* < 0.05).

## 3. Discussion

With the large-scale and industrial development of *C. nasus* aquaculture, ill-matched feed compositions severely affect the growth performance, health status, and even survival rates of the fish. Carbohydrates and fats, as the primary forms of energy storage and supply, play a crucial role in the life activities of fish. One of the most common consequences of disrupted carbohydrate and lipid metabolism is liver damage [23,24]. For instance, largemouth bass are prone to developing fatty liver under high-sugar diets. Fatty liver not only affects the growth and development of fish but also leads to severe health issues, such as liver fibrosis and liver function failure [25]. Carbohydrate and lipid metabolic disorders can also lead to insulin resistance and glucose metabolic disorders in fish. For example, Nile tilapia fed a high-monosaccharide diet exhibit increased insulin levels and abnormal blood glucose levels. This is due to the impairment of insulin signaling pathways, which prevents glucose from entering cells normally, further exacerbating fluctuations in blood glucose concentrations [26]. Prolonged carbohydrate and lipid metabolic disorders can lead to growth inhibition and a decline in immune function in fish [27,28]. In this study, high glucose and high lipid treatments significantly increased glucose, triglyceride, and cholesterol levels in *C. nasus* hepatocytes. Activities of ALT, AST and MDA concentration increased, while activities of CAT and SOD decreased. This suggested that these treatments caused carbohydrate and lipid metabolic disorders in hepatocytes, reducing their antioxidant capacity and causing damage.

SCFAs, also known as volatile fatty acids, are organic linear carboxylic acids with fewer than six carbons, including acetic acid, propionic acid, butyric acid, and others [29,30]. Apart from a small fraction obtained directly from food, the majority of SCFAs are produced by the anaerobic fermentation of gut microbiota [31]. In aquatic animals, acetic acid, propionic acid, and butyric acid are the most predominant SCFAs [32], playing a crucial role in regulating host metabolism, immune system, and cell proliferation [33,34]. SCFAs are absorbed into the cytoplasm of enterocytes in the gut, where they participate in the host’s energy metabolism, particularly in glucose and lipid metabolism. Studies have shown that SCFAs are involved in the regulation of glucose and lipid homeostasis [35]. In this study, co-incubating hepatocytes of *C. nasus* with acetic acid and propionic acid in the presence of high glucose and high lipid conditions led to a significant reduction in triglyceride and cholesterol levels in the hepatocytes. The addition of appropriate amounts of sodium acetate, sodium propionate, and sodium butyrate to the feed can promote the growth of species such as the Chinese sturgeon, Caspian whitefish, and grass carp, and reduce blood glucose, triglyceride, and cholesterol levels [4,5,6]. Nevertheless, co-incubating hepatocytes of *C. nasus* with acetic acid and propionic acid in the presence of high glucose and high lipid conditions led to a significant increase in glucose levels in the hepatocytes. Under hyperglycemic conditions, short-chain fatty acids (SCFAs) demonstrate dual regulatory effects on glucose homeostasis. Specifically, they facilitate cellular glucose uptake while simultaneously suppressing hepatic gluconeogenesis and enhancing peripheral glucose utilization, thereby collectively contributing to blood glucose reduction [36]. This coordinated mechanism may elucidate the observation in our study where acetate and propionate administration resulted in transient glucose accumulation within hepatocytes, potentially reflecting a compensatory metabolic adaptation during acute phase regulation. Furthermore, acetate and propionate acids significantly increased the activities of antioxidant enzymes CAT and SOD and significantly reduced the levels of MDA. High glucose can affect the antioxidant capacity of fish, leading to oxidative stress. Safari et al. (2017) found that dietary supplementation with sodium propionate enhances the antioxidant capacity of carp and has a beneficial effect on the antioxidant defense system [37]. The addition of sodium propionate to the feed can enhance the activity of catalase and glutathione peroxidase in the Chinese sturgeon and significantly reduce the content of malondialdehyde [4]. These findings indicate that acetic acid and propionic acid can significantly improve the glucose and lipid levels in hepatocytes of *C. nasus* treated with high glucose and high lipids and enhance the antioxidant capacity to combat the oxidative stress induced by high glucose and high lipids.

The liver plays a crucial role in maintaining glucose homeostasis. GCK plays a significant role in the cellular uptake and utilization of glucose [38]. PFK, as a key enzyme in the glycolytic pathway, is involved therein [39]. G6PC is a key enzyme in maintaining glucose homeostasis and is involved in gluconeogenesis. PEPCK is the rate-limiting enzyme in hepatic gluconeogenesis [40]. In this study, the addition of acetate and propionate acids significantly increased the expression of glycolysis-related genes gck and pfk while significantly decreasing the expression of *g6pca1*, *g6pca2*, and *pck*, indicating that they promoted glycolysis and inhibited gluconeogenesis in the hepatocytes of *C. nasus*. It has found that acetic acid can promote glucose uptake in astrocytes, enhance the activity of the key glucose metabolic enzyme pyruvate carboxylase (PC), and thereby promote glycolysis and the TCA cycle [41]. Furthermore, sodium acetate directly reduces the expression of G-6-Pase and PEPCK in the liver by activating the AMPK-GPR43 pathway, inhibiting the process of gluconeogenesis, thereby lowering blood glucose levels [42]. The addition of sodium propionate to the diet can reduce the expression of G-6-Pase and PEPCK in the liver of carp, thereby inhibiting gluconeogenesis; sodium propionate can also increase the expression of PK and PFK, promoting glycolysis. As a crucial organ for regulating lipid metabolism in animals, the liver plays an essential role in maintaining lipid metabolic balance. SREBP1 plays a key role in lipid metabolism, including the synthesis of cholesterol, fatty acids, and phospholipids [43]. ACC regulates the rate-limiting step of fatty acid biosynthesis [44]. DGAT is crucial for fat absorption and storage. ELOVL6 is an enzyme involved in fatty acid elongation and synthesis [45]. HSL hydrolyzes triglycerides and plays an irreplaceable role in lipolysis. PPARα is involved in fatty acid oxidation [46]. Carnitine palmitoyltransferase 1 (CPT1) is responsible for the translocation of long-chain fatty acids from the cytoplasm to the mitochondria for β-oxidation [47]. ACADM (acyl-CoA dehydrogenase, medium chain) participates in the β-oxidation of fatty acids. In this study, the addition of acetic and propionic acids significantly downregulated the expression of *srebp1*, *acc*, *dgat*, and *elovl6* in the hepatocytes of *C. nasus*, while significantly upregulating the expression of *hsl*, *pparα*, *cpt1*, and *acadm*, indicating that acetic and propionic acids promoted fatty acid oxidation and catabolism while inhibiting lipid synthesis in the hepatocytes of *C. nasus*. The sodium acetate, by upregulating the expression of genes related to lipolysis and fatty acid oxidation, reduces the accumulation of triglycerides and helps prevent the deposition of fat in the liver [48]. Furthermore, in the HepG2 cell model, sodium propionate significantly increased the expression of genes such as ATGL, CPT1α, and FGF21, promoting fatty acid oxidation and reducing triglyceride storage [48]. Sodium acetate and sodium propionate significantly increased the expression of PPARα, thereby regulating genes related to cholesterol metabolism [48]. These results indicated that acetic acid and propionic acid regulated glucose metabolism in hepatocytes of *C. nasus* by promoting glycolysis and inhibiting gluconeogenesis; they also regulated lipid metabolism by inhibiting lipid synthesis and promoting lipid catabolism, thereby ameliorating glucose and lipid metabolic disorders and cell damage induced by high glucose and high lipids in hepatocytes of *C. nasus*.

**Figure 9:**
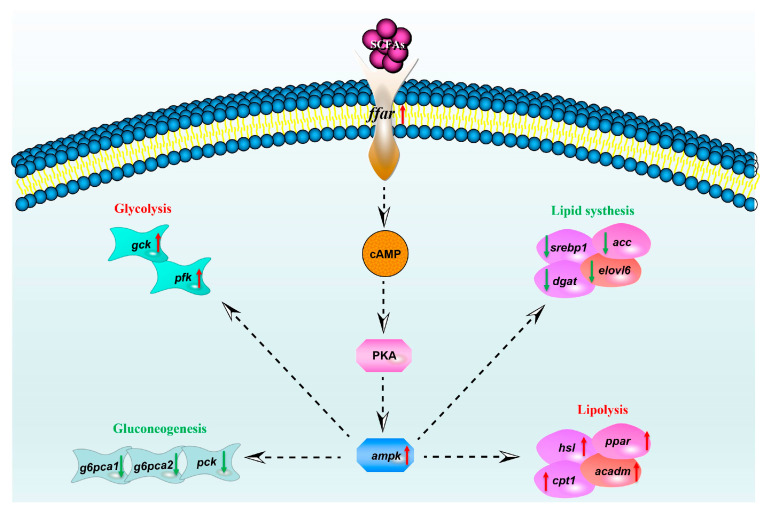
Hypothetical model of SCFA-mediated regulation of glucose–lipid metabolism in *C. nasus* hepatocytes. Dotted lines indicated potential regulatory pathways. Red arrows indicated upregulation, and green arrows indicated downregulation.

In aquatic animals, short-chain fatty acids (SCFAs) play a crucial role in improving growth performance, regulating glucose and lipid metabolism, and alleviating intestinal inflammation. As small molecular substances, the receptors through which SCFAs activate intracellular cascades have not yet been reported in studies. In higher animals, SCFAs can trigger cell-specific signal cascades by activating G protein-coupled free fatty acid receptors FFAR2 (GPR43) and FFAR3 (GPR41) [49,50]. FFAR2 and FFAR3 are both associated with metabolic diseases and have become effective targets for the treatment of type 2 diabetes, asthma, cardiovascular diseases, and metabolic syndrome [51,52]. To characterize and elucidate the regulatory mechanisms of SCFAs on glucose and lipid metabolism, this study employed molecular docking simulations to analyze the binding affinity of acetic acid and propionic acid with *C. nasus,* FFAR2 and FFAR3. The results indicated that the binding energies of acetic acid with *C. nasus* FFAR2 and FFAR3 were −8.18 kcal/mol and −9.06 kcal/mol, respectively. The binding energies of propionic acid with *C. nasus* FFAR2 and FFAR3 were −8.39 kcal/mol and −9.52 kcal/mol, respectively. A lower binding energy indicates a more stable ligand-receptor complex, and the minimum binding energies of the target proteins FFAR2 and FFAR3 with acetic acid and propionic acid were less than −5.0 kcal/mol, suggesting potential binding activity between the ligands and receptors. In humans, the affinity ranking of FFAR2 for SCFAs is acetic acid = propionic acid > butyric acid > valeric acid, while the affinity ranking for FFAR3 is propionic acid = butyric acid = valeric acid > acetic acid [49,50]. This is consistent with the results of this study. Moreover, acetic and propionic acids increased expression of *ffar2*, *ffar3*, and *ampkα*. In mammalian systems, after binding to FFAR2/3, SCFAs are known to activate downstream signaling molecules such as adenylyl cyclase and phospholipase C through G protein coupling, which can lead to increased intracellular cAMP levels and calcium ion influx [53,54]. In these models, elevated cAMP levels typically activate PKA, a kinase that may phosphorylate AMPK to modulate enzymes related to fatty acid oxidation and lipid synthesis [55,56]. Furthermore, AMPK activation has been associated with reduced hepatic gluconeogenesis through the phosphorylation of key enzymes such as PEPCK and G6Pase [47]. Our observation of SCFA-induced normalization of glucose and lipid metabolism, combined with potential interactions between SCFA and FFAR2/3, suggested the possibility that acetate and propionate might regulate glucose-lipid metabolism in *C. nasus* hepatocytes through mechanisms potentially involving FFAR-associated signaling and AMPK-related responses. This hypothetical pathway integration is depicted in Figure 9 as a dashed-line model aligning with teleost-specific signaling characteristics. However, this model required direct validation, such as direct quantification of phosphorylated AMPK by Western blot, loss-of-function (e.g., CRISPR/Cas9 knockout or pharmacological inhibition) validation between SCFAs and FFAR, as well as AMPK and downstream genes.

## 4. Materials and Methods

### 4.1. Primary Culture of C. nasus Hepatocytes

The primary hepatocyte culture of *C. nasus* was conducted with reference to [57,58]. Select healthy *C. nasus* and euthanize after blood collection under anesthesia. Disinfect with a 70% ethanol solution (Solarbio, Beijing, China) and place on a sterile workbench. Aseptically dissect liver tissue and place it into pre-chilled 1 × PBS (Solarbio, Beijing, China) containing double antibiotics (200 IU) (Solarbio, Beijing, China). Remove fascia, blood clots, and other tissues, and rinse with PBS 2–3 times. Place the processed tissue in a sterile culture dish and finely mince the tissue. Add 5–10 volumes of 0.25% trypsin (Merck, Shanghai, China) and digest at room temperature (25–28 °C) for 10–15 min. Terminate the digestion by adding an appropriate amount of L-15 medium (Merck, Shanghai, China) containing 10% fetal bovine serum. After digestion, pass the mixture through a 200-mesh nylon sieve, triturate, and centrifuge at 1000 rpm for 5 min, discarding the supernatant. Resuspend the tissue cells in basal medium, centrifuge at 1000 rpm for 5 min to remove the supernatant, and resuspend the sedimented cells in L-15 medium containing 10% fetal bovine serum (Merck, Shanghai, China). Count the cells and adjust the cell concentration to 10^6^ cells/mL. After plating, culture in a cell incubator at 28 °C with 5% CO_2_.

### 4.2. Construction of High Glucose and High Lipid Models

Collect cells in the logarithmic growth phase and adjust the density to 1 × 10^6^ cells/mL. Seed the primary hepatocytes of *C. nasus* into a 96-well plate at 100 μL per well. Set up six different concentrations of glucose (0, 5, 10, 15, 20, and 25 mmol/L) [59,60] obtained from Merck (Shanghai, China) to incubate the hepatocytes, with four replicates for each group, and collect the cells after 24 h. Additionally, set up six different concentrations of oleic acid (0, 0.2, 0.4, 0.6, 0.8, and 1.0 mmol/L) [61] obtained from Merck (Shanghai, China) to incubate the hepatocytes, with four replicates for each group, and collect the cells after 24 h.

### 4.3. SCFA Incubation Treatment

Collect cells in the logarithmic growth phase and adjust the density to 1 × 10^6^ cells/mL. Seed the primary hepatocytes of *C. nasus* into a 96-well plate at 100 μL per well. Set up five different concentrations of acetic acid and propionic acid (0, 2, 4, 6, and 8 mmol/L) [62] obtained from Merck (Shanghai, China) to co-incubate with hepatocytes in the presence of glucose (25 mmol/L) and oleic acid (1.0 mmol/L). Each group contains four replicates, and the cells are collected after 24 h of incubation.

### 4.4. Cell Viability Assay

Cell viability was assessed using the CCK-8 method. Add 10 μL of CCK-8 (Merck, Shanghai, China) solution to each well, and incubate in the incubator for 1 h. After incubation, measure the absorbance of each group’s samples at 450 nm using a microplate reader. The calculation method is as follows: cell viability (%) = [A (treated) − A (blank)]/[A (control) − A (blank)].

### 4.5. Detection of Glucose, Triglycerides, Cholesterol, and Immunoenzyme Activity

The activities of catalase (CAT), superoxide dismutase (SOD), alanine aminotransferase (ALT), and aspartate aminotransferase (AST), as well as the concentrations of cholesterol (CHO), low-density lipoprotein cholesterol (LDL-C), and malondialdehyde (MDA) were detected using assay kits purchased from Nanjing Jincheng Bioengineering Institute (Nanjing, China). The assay kits for glucose (GLU) and triglyceride (TG) concentrations were obtained from Beijing Solaibao Biotechnology Co., Ltd. (Beijing, China). The specific experimental procedures were carried out according to the instructions provided with the respective kits.

### 4.6. RT-qPCR Analysis of Hepatic Glucose and Lipid Metabolism Gene Expression

The RNA was extracted by RNA isolater Total RNA Extraction Reagent (Vazyme, Nanjing, China). The first strand of gDNA was synthesized by HiFiScript gDNA Removal RT MasterMix (Cowin Biosciences, Taizhou, China) according to the manufacturer’s instructions. Primers for RT-qPCR were designed based on the genomic CDS sequences of *C. nasus* (GenBank GCA_007927625.1) using Primer Premier 5 software (see Supplementary Table S1). The qPCR was performed on a Bio-Rad CFX96 real-time PCR system (Bio-Rad, Hercules, CA, USA) using a total 20.0 μL reaction system including 10.0 μL ChamQ Universal SYBR qPCR Master Mix (Vazyme, Nanjing, China), 1.0 μL gDNA template, 1.0 μL each primer (10 μmol/L), and 7.0 μL PCR-grade DEPC water. The reaction system (20.0 μL) included 10.0 μL of iTaqTM Universal SYBR^®^ Green Supermix (Bio-Rad), 2.0 μL of cDNA, 1.0 μL of each primer (10 μmol/L), and 6.0 μL of PCR-grade DEPC water. Reactions were performed in triplicate per sample, and cycling parameters were set as follows: 94 °C for 2 min, followed by 40 cycles of 15 s at 94 °C, 30 s at 60 °C, and 45 s at 72 °C. *β-actin* was used as the reference gene. All samples were analyzed in triplicate, and the expression levels of the target genes were calculated using the 2^−ΔΔCT^ method.

### 4.7. Data Analysis

All results are presented as means ± standard error (SE). If the data of cell viability and acetic acid and propionic acid treatment obeyed normalized distribution (Kolmogorov–Smirnov test) and variance homogeneity (Levene test), differences between different temperature-treated groups were determined using one-way ANOVA and Duncan’s multiple-range test using SPSS 20.0. Otherwise, a nonparametric test (Kruskal–Wallis test) was used to complete the analysis of variance. Normal distribution of data was tested with Shapiro–Wilk (α = 0.05). Results of high glucose and high lipid models were tested using Student’s *t*-test using SPSS 20.0. The significance level was set at *p* < 0.05 to indicate statistical significance. Bar graphs were generated using GraphPad Prism 10.0.

### 4.8. Molecular Docking

Homology modeling of the FFAR2 and FFAR3 from *C. nasus* was performed using the SwissModel server (https://swissmodel.expasy.org/). To identify FFAR2 and FFAR3 sequences in *C. nasus*, the whole genome databases (GenBank GCA_007927625.1) were searched using BLAST GUI Wrapper on TBtools (v1.0692) according to amino sequences of human (Homo sapiens), zebrafish, and Atlantic herring (*Clupea harengus*) downloaded from Ensembl (http://www.ensembl.org) and NCBI (http://www.ncbi.nlm.nih.gov/) databases (cutoff value < 1e−5). After removing repeated sequences, the unique sequences were validated via BLASTN against the NCBI nonredundant protein database. The pKa values of amino acids under neutral conditions were calculated and assigned using the online tool propka3 (https://www.ddl.unimi.it/vegaol/propka.htm) (accessed on 15 August 2024). The receptor and ligand structures for docking were prepared using Autodock Tools-1.5.7. Molecular docking experiments were conducted with the Watvina software (https://github.com/biocheming/watvina) (accessed on 15 August 2024), setting the box size to a cube with an edge length of 126 Å (semi-flexible global docking), and the spacing step size was set to 0.375. The maximum number of conformations to search was set to 10,000, and a genetic algorithm was used for conformation sampling and scoring. The conformations were ranked, and the optimal conformation was selected based on the docking scores.

## 5. Conclusions

Acetate and propionate acids mitigated metabolic disorders induced by high glucose and lipids by enhancing antioxidant enzyme activity and modulating glucose and lipid metabolic pathways. Molecular docking indicates that acetic and propionic acids bind effectively to *C. nasus* FFAR 2 and 3, suggesting that their beneficial effects are mediated by receptor activation and subsequent cellular signaling cascades. This study provided new insights for the development of novel feed additives and the prevention and treatment of metabolic diseases in aquatic animals and deepened the understanding of the functions of SCFAs in nonmammalian vertebrates as well as the interaction mechanisms between gut microbiota and host metabolism.

## Data Availability

No new data were created or analyzed in this study. Data sharing is not applicable to this article.

## Supplementary Materials

The following supporting information can be downloaded at https://www.mdpi.com/article/10.3390/ijms26083654/s1.
