# Supplementary material for: Short-Chain Fatty Acids (SCFAs) Modulate the Hepatic Glucose and Lipid Metabolism of Coilia nasus via the FFAR/AMPK Signaling Pathway In Vitro
Source: Int J Mol Sci. 2025 Apr 12;26(8):3654. doi: 10.3390/ijms26083654 (PMC12027798; doi:10.3390/ijms26083654)
Supplement: Supplementary file 1 [file ijms-26-03654-s001.zip › Supplementary Files/Supplementary_Figure.pdf]

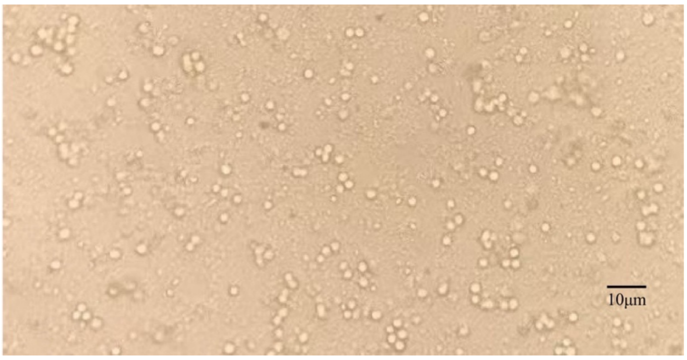

Figure S1. Morphology of *C. nasus* hepatocytes (× 100).

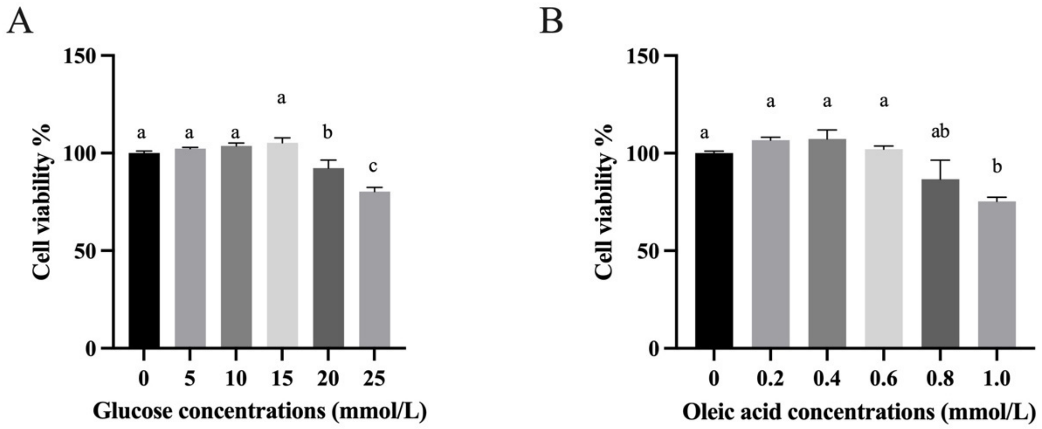

Figure S2. Effects of high glucose (A) and high lipid (B) on the cell viability of *C. nasus* hepatica. Different letters indicate a significant difference ($P < 0.05$).

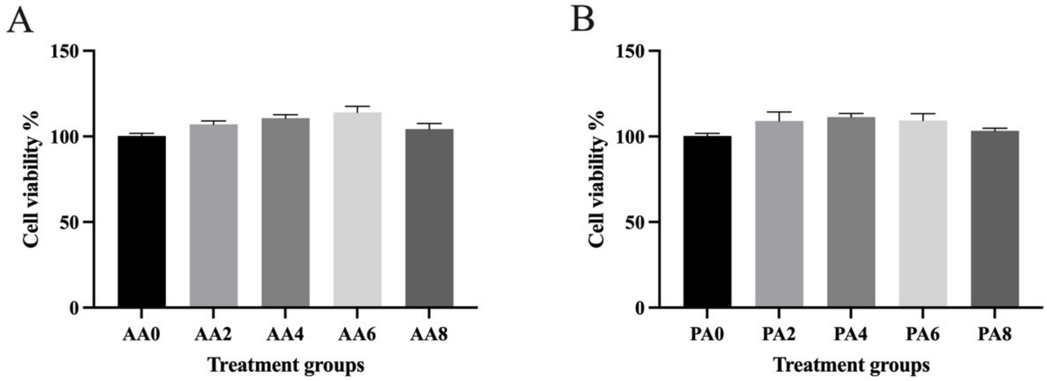

Figure S3. Effects of acetate acid (A) and propionate acid (B) on cell viability of *C. nasus* hepatocytes treated with high glucose.

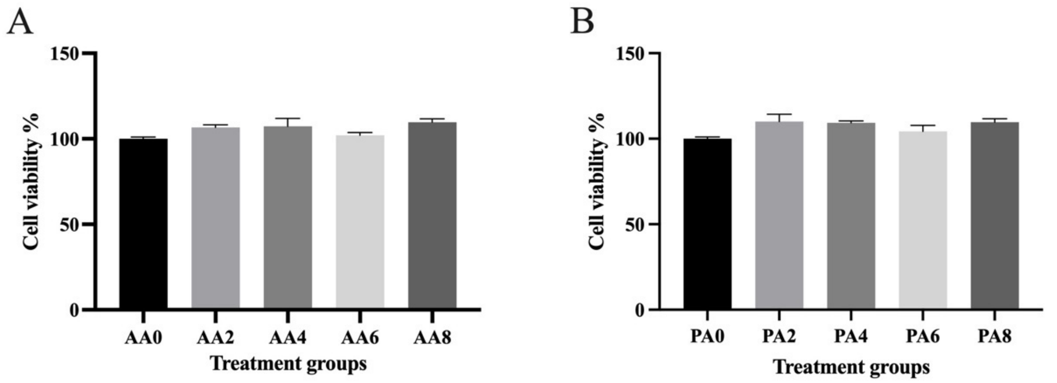

Figure S4. Effects of acetate acid (A) and propionate acid (B) on cell viability of *C. nasus* hepatocytes treated with high lipid.

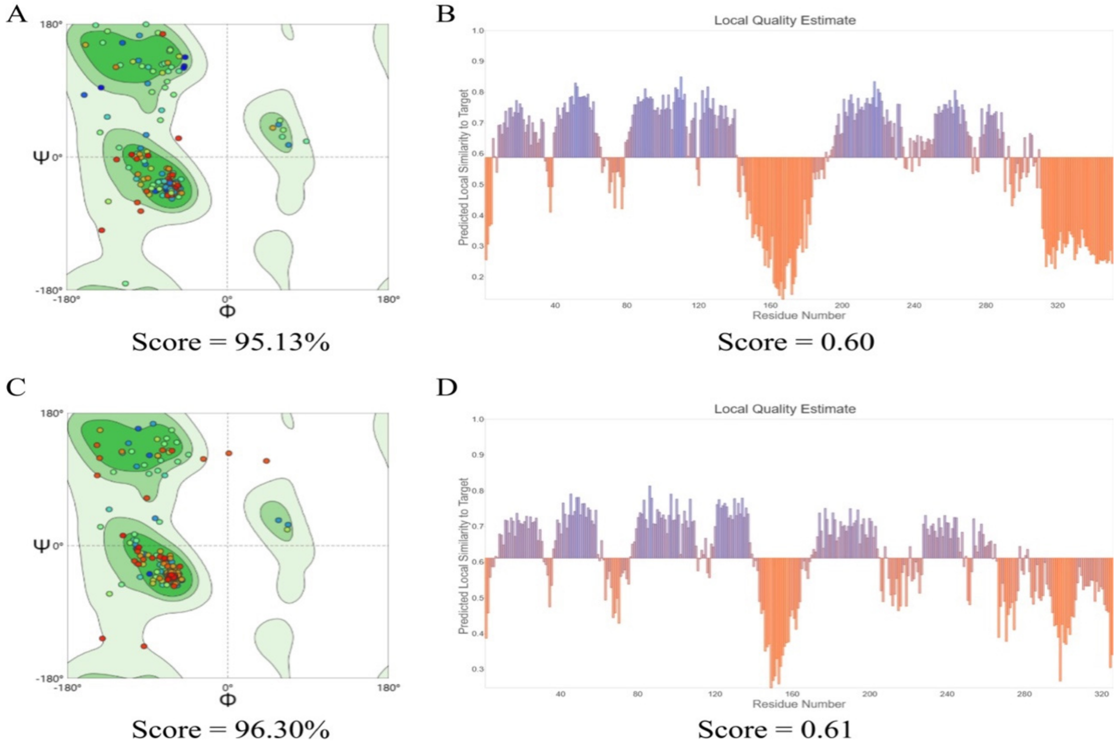

Figure S5. Homologous modeling results of FFAR2 and FFAR3. Ramachandran Plot score of FFAR2 (A), FFAR2 QMEAN local scores (B); Ramachandran Plot score of FFAR3 (C), FFAR3 QMEAN local scores (D).
